# Alzheimer’s Disease Risk Variant rs3865444 in the *CD33* Gene: A Possible Role in Susceptibility to Multiple Sclerosis

**DOI:** 10.3390/life12071094

**Published:** 2022-07-21

**Authors:** Juraj Javor, Mária Bucová, Vladimíra Ďurmanová, Dominika Radošinská, Zuzana Párnická, Daniel Čierny, Egon Kurča, Daniela Čopíková-Cudráková, Karin Gmitterová, Ivana Shawkatová

**Affiliations:** 1 Institute of Immunology, Faculty of Medicine, Comenius University in Bratislava, 811 08 Bratislava, Slovakia; maria.bucova@fmed.uniba.sk (M.B.); vladimira.durmanova@fmed.uniba.sk (V.Ď.); dominika.radosinska@fmed.uniba.sk (D.R.); zuzana.parnicka@fmed.uniba.sk (Z.P.); ivana.shawkatova@fmed.uniba.sk (I.S.); 2 Department of Clinical Biochemistry, Jessenius Faculty of Medicine in Martin, Comenius University in Bratislava and University Hospital Martin, 036 59 Martin, Slovakia; daniel.cierny@uniba.sk; 3 Clinic of Neurology, Jessenius Faculty of Medicine in Martin, Comenius University in Bratislava and University Hospital Martin, 036 59 Martin, Slovakia; egon.kurca@uniba.sk; 4 1st Department of Neurology, Faculty of Medicine, Comenius University in Bratislava and University Hospital Bratislava, 813 69 Bratislava, Slovakia; dcudrakova@zoznam.sk; 5 2nd Department of Neurology, Faculty of Medicine, Comenius University in Bratislava and University Hospital Bratislava, 833 05 Bratislava, Slovakia; karin.gmitterova@fmed.uniba.sk

**Keywords:** association, CD33, multiple sclerosis, polymorphism, rs3865444, severity, susceptibility

## Abstract

Polymorphisms in genes encoding receptors that modulate the activity of microglia and macrophages are attractive candidates for participation in genetic susceptibility to multiple sclerosis (MS). The aims of the study were to (1) investigate the association between Alzheimer’s disease-linked variant rs3865444:C>A in the *CD33* gene and MS risk, (2) assess the effect of the strongest MS risk allele HLA-DRB1*15:01 on this association, and (3) analyze the correlation of rs3865444 with selected clinical phenotypes, i.e., age of onset and disease severity. *CD33* rs3865444 was genotyped in a cohort of 579 patients and 1145 controls and its association with MS risk and clinical phenotypes was analyzed by logistic and linear regression analysis, respectively. Statistical evaluation revealed that rs3865444 reduces the risk of MS in the HLA-DRB1*15:01-positive subpopulation but not in the cohort negative for HLA-DRB1*15:01. A significant antagonistic epistasis between rs3865444 A and HLA-DRB1*15:01 alleles in the context of MS risk was detected by the interaction synergy factor analysis. Comparison of allele and genotype distribution between relapsing-remitting MS, secondary progressive MS, and control groups revealed that rs3865444 C to A substitution may also be associated with a decreased risk of transition of MS to its secondary progressive form, irrespective of the HLA-DRB1*15:01 carrier status. On the other hand, no correlation could be found between rs3865444 and the age of disease onset or MS severity score. Future studies are required to shed more light on the role of CD33 in MS pathogenesis.

## 1. Introduction

Multiple sclerosis (MS) is an autoimmune disease of the central nervous system (CNS) characterized by multifocal chronic inflammation, loss of oligodendrocytes, gliosis, and demyelination that leads to neuro-axonal degeneration and progressive neurological disability [1]. The exact cause of MS remains unknown, but complex interactions between genetic and environmental factors are at play [2]. Genome-wide association studies (GWAS) have greatly improved our understanding of MS pathogenesis by identifying numerous small-effect risk loci that form the genetic architecture of the disease [3]. The most recent and largest GWAS to date discovered 233 robustly associated susceptibility variants, which together with additional suggestive loci, explain up to 48% of the overall heritability of MS [4]. Additional analyses observed enrichment for MS susceptibility loci in different cells of the peripheral and brain-resident immune systems, including B and T cells, natural killer cells, myeloid cells, and microglia, emphasizing the role of both the adaptive and innate arms of immunity in MS development [4].

MS has been traditionally considered to develop as a result of autoreactive T and B cells migrating from the periphery and targeting myelin antigens in the CNS [2,5]. Recent studies have begun to highlight the key role of microglia and monocyte-derived macrophages (MDM), which are abundant in demyelinating lesions in the brain and the spinal cord [6,7]. These cells exhibit dynamic phenotypic plasticity and, depending on context and their activation state, may have both beneficial and detrimental roles in MS pathogenesis [5,8,9,10]. Their neurotoxic action is linked to the production of pro-inflammatory cytokines, reactive oxygen and nitrogen species, and the presentation of myelin-derived antigens to T cells, ultimately contributing to demyelination and axonal loss. The neuroprotective effects include removal of myelin debris and apoptotic cells by phagocytosis, release of anti-inflammatory molecules, and stimulation of the proliferation and growth of oligodendrocytes and their progenitor cells, all of which are essential for resolving inflammation, promoting remyelination, and preventing axonal degeneration [9,11,12,13]. Dysregulation of microglial/macrophage activation with a shift towards the neurotoxic phenotype can significantly disrupt the remyelination process that takes place in the early phases of MS, thereby contributing to disease development and progression [9,14,15].

The phagocytic uptake and clearance of protein aggregates and debris are under the control of several different membrane receptors, including the scavenger and pattern-recognition receptors, such as CD36, triggering receptor expressed on myeloid cells 2 (TREM2), sialic acid-binding immunoglobulin-type lectins (Siglecs), and others [8,16,17,18]. Their gene polymorphisms are therefore attractive candidates for participation in genetic susceptibility to MS. Recently, an association between the single nucleotide polymorphism (SNP) rs3865444:C>A in the *CD33* gene and MS was reported in the Greek population [19]. Interestingly, rs3865444 is also one of the top-ranked Alzheimer’s disease (AD) risk variants [20,21,22,23], indicating that some individual neuroinflammatory mechanisms are shared by different neurological diseases such as MS and AD [15]. *CD33* is located on chromosome 19q13.41 and encodes a 67-kDa transmembrane receptor CD33, also known as Siglec-3, expressed mainly on cells of the myeloid lineage, including granulocytes, microglia, monocytes, macrophages, and dendritic cells [24]. Its extracellular ligand-binding Ig V-set domain recognizes sialic acid-containing glycoproteins and glycolipids on the surface of mammalian cells, triggering inhibitory signaling involved in the regulation of myeloid cell function and activities [24,25,26]. The rs3865444 SNP located upstream of the *CD33* transcription start site is indirectly associated with increased production of alternatively spliced CD33 isoform (CD33m, D2-CD33) lacking the Ig V-set domain at the expense of the full-length CD33M [27,28,29,30,31,32,33,34,35], resulting in alterations of microglial and macrophage functions [36,37,38,39].

Independent replication remains an important tool for understanding the role of genetic risk factors across different populations and ethnicities. Given that the role of *CD33* rs3865444 in MS susceptibility was reported only in a single paper [19], we decided to perform a case-control study to evaluate the impact of this SNP on MS risk in the Slovak population and further examine whether its association with the disease is affected by the HLA-DRB1*15:01 allele as the single strongest MS genetic risk factor [40,41]. In addition to susceptibility, genetic determinants may also influence various clinical phenotypes [3]. Therefore, we also analyzed the effect of rs3865444 on the age of MS onset and disease severity.

## 2. Materials and Methods

### 2.1. Study Subjects

A total of 1724 Slovak Caucasian subjects were recruited for the purposes of an ongoing study of MS risk factors. The MS patient group consisted of 579 unrelated individuals (403 females and 176 males) recruited at the neurology departments of university hospitals in Bratislava and Martin, Slovakia. The diagnosis of MS was based on the 2010 revised McDonald criteria [42], and only patients with a relapse-onset form of the disease, namely relapsing-remitting (RR) or secondary progressive (SP) MS, were included in the study. The age of onset (AOO) was defined by the first episode of neurological dysfunction suggestive of CNS demyelinating disease. The degree of patients’ neurological disability at the time of examination was determined using Kurtzke’s Expanded Disability Status Scale (EDSS) [43], which was subsequently used to assess the Multiple Sclerosis Severity Score (MSSS) as a measure of the rate of disability accumulation and disease severity. For this purpose, a table with global MSSS generated from 9892 European patients was employed, and the score of each individual patient was simply ascertained by finding the column corresponding to the patient’s EDSS and the row corresponding to the number of years since the onset of MS [44]. The control group comprised 1145 unrelated adults (717 females and 428 males) without a personal or family history of MS and other common autoimmune and neurological diseases. The basic demographic and clinical characteristics of patients and controls are summarized in Table 1.

Written informed consent for the enrolment in the study and for personal data management was obtained from all study participants. The investigations were carried out in accordance with the International Ethical Guidelines and the World Medical Association Declaration of Helsinki. The study was approved by the Independent Ethical Committee of the University Hospital Bratislava and the Faculty of Medicine, Comenius University in Bratislava.

### 2.2. Genotyping

Genomic DNA was extracted from EDTA-treated blood samples using the standard phenol-chloroform method. Genotyping of *CD33* rs386544 as well as of the specific HLA-DRB1*15:01-tagging SNP rs3135388 [45,46] was performed by the polymerase chain reaction-restriction fragment length polymorphism method according to previously published protocols [47,48]. For quality control, 10% of samples were randomly selected and genotyped in duplicate, and several cases of each genotype were confirmed by direct DNA sequencing using the BigDye^®^ Terminator v3.1 Cycle Sequencing Kit and Applied Biosystems 3130xl Genetic Analyzer (Life Technologies, Carlsbad, CA, USA). The reproducibility of the results was 100%.

### 2.3. Statistical Analysis

Differences in categorical variables (sex, MS type, HLA-DRB1*15:01 carrier status) between the study groups were evaluated by the χ^2^ test, whereas continuous variables (age, AOO, disease duration, EDSS, MSSS) were compared by the Welch’s corrected t-test (for normally distributed data) or Mann–Whitney test (for nonparametric data). Positive HLA-DRB1*15:01 carrier status was defined as the presence of at least one copy of rs3135388 T allele tagging the HLA-DRB1*15:01 allele. The statistical power of our case-control study was calculated using the online web tool Genetic Association Study (GAS) Power Calculator (https://csg.sph.umich.edu/abecasis/gas_power_calculator/index.html, accessed on 13 July 2022). *CD33* rs3865444 genotypes were tested for possible departure from Hardy–Weinberg equilibrium (HWE) by the χ^2^ goodness-of-fit test with 1 degree of freedom. The association between rs3865444 and MS risk was examined by the logistic regression analysis adjusted for age, sex, and HLA-DRB1*15:01 carrier status as possible confounding covariates. *p*, odds ratio (OR), and 95% confidence interval (CI) values were computed for the effects of alleles or genotypes in allelic, codominant, dominant, recessive, over-dominant, and log-additive inheritance models. Regression analysis and synergy factor (SF) measurement were used to assess the significance and size of the interaction between *CD33* rs3865444 A and HLA-DRB1*15:01 alleles, as previously described [49]. SF is defined as the ratio of the observed OR for both factors combined (OR_12_) to the predicted OR assuming independent effects of each factor (OR_1_ × OR_2_). The correlation of *CD33* rs3865444 genotypes with AOO and MSSS was tested using the linear regression analysis. *p*-values < 0.05 obtained in the above mentioned statistical tests were considered statistically significant. The analyses were performed with the InStat statistical software package (GraphPad Software, Inc., San Diego, CA, USA) and the SNPStats web software available at http://bioinfo.iconcologia.net/SNPstats, accessed on 14 April 2022 [50].

## 3. Results

### 3.1. Characteristics of Study Subjects

A total of 579 patients diagnosed with MS were enrolled in this study, including 403 (69.6%) females and 176 (30.4%) males. The mean age was 41.6 years, the mean age of disease onset was 29.7 years, and the mean duration of MS was 11.9 years. The comparison of basic clinical parameters did not reveal any significant differences between male and female MS patients (Table 1). The control group comprised 1145 unrelated individuals with a mean age of 51.9 years, out of whom 717 (62.6%) were females and 428 (37.4%) were males. As shown in Table 1, the mean age of controls was significantly higher (*p* < 0.0001) and their female-to-male ratio was lower (*p* = 0.0041) compared to MS patients. Consistent with our previous observations [51,52], carriers of at least one copy of the major MS risk allele HLA-DRB1*15:01 were significantly overrepresented in the MS group compared to controls (51.5% vs. 20.1%; *p* < 0.0001). As shown in Supplementary Table S1, one copy of the HLA-DRB1*15:01 allele was associated with almost 4-fold increased odds of MS (OR = 3.77; 95% CI = 2.98–4.76; *p* < 0.0001) while subjects homozygous for the allele had 17-fold increased odds of developing the disease (OR = 17.01; 95% CI = 7.73–37.39; *p* < 0.0001). Due to their different distribution between the patient and control groups, HLA-DRB1*15:01 carrier status, age, and sex were included in *CD33* rs3865444 association analyses as possible confounding factors.

### 3.2. Association of CD33 rs3865444 with MS Risk

The statistical power of our case-control study was estimated using the online GAS Power Calculator. With 579 cases and 1145 controls, and assuming the MS prevalence of 0.1%, minor allele frequency of 31%, and an additive model, the power of our study to detect an association at a significance level of 0.05 was 88% for a relative risk equal to 1.3 but only 22% for a relative risk of 1.1.

The genotype distribution of *CD33* rs3865444 did not show any significant departure from HWE in MS patients (χ^2^ = 0.78, *p* = 0.38) or controls (χ^2^ = 0.62, *p* = 0.43). Analysis of rs3865444 allele frequencies in study cohorts revealed no significant difference between MS patients and controls (allele A: 30.4% vs. 31.1%; *p* = 0.68; OR = 0.97; 95% CI = 0.83–1.13). In line with this finding, logistic regression analysis adjusted for the HLA-DRB1*15:01 carrier status, age, and sex did not find an association of *CD33* rs3865444 with MS risk in any of the genetic models (Table 2).

To explore the possible effect of the HLA-DRB1*15:01 allele on the association between rs3865444 and MS, we next examined allele and genotype distribution in cohorts stratified according to the HLA-DRB1*15:01 carrier status. Logistic regression analysis revealed an association of the rs3865444:C>A substitution with a reduced risk of MS in the HLA-DRB1*15:01-positive subpopulation under codominant (*p* = 0.028, OR = 0.64, 95% CI = 0.44–0.95), dominant (*p* = 0.040, OR = 0.68, 95% CI = 0.47–0.98), and over-dominant models (*p* = 0.031, OR = 0.66, 95% CI = 0.46–0.96), while no significant effect of rs3865444 on MS risk was found in the subpopulation negative for the HLA-DRB1*15:01 allele (Table 3).

To further evaluate the statistical epistasis between *CD33* rs3865444 A and HLA-DRB1*15:01 alleles, we performed an interaction SF analysis and assessed the risk of developing MS in subjects carrying either one of these traits or both when compared to subjects negative for both alleles. As shown in Table 4, the observed combined effect size of the two alleles (OR = 3.67) was lower than the predicted joint OR assuming independent effects of both rs3865444 A and HLA-DRB1*15:01 (OR = 5.93). As a result, the calculated SF value of 0.62 significantly deviated from 1 (*p* = 0.032), suggesting that *CD33* rs3865444 A and HLA-DRB1*15:01 alleles displayed an antagonistic statistical interaction in the context of MS risk. On the other hand, no such interaction was observed between the *CD33* rs3865444 A allele and sex (*p* = 0.68).

Next, we compared the *CD33* rs3865444 allele and genotype distribution between RR-MS, SP-MS, and control groups. As shown in Table 5, carriers of the minor A allele were significantly underrepresented in the SP-MS group compared to both the RR-MS (dominant model: *p* = 0.0023, OR = 0.41, 95% CI = 0.23–0.74) and control groups (dominant model: *p* = 0.0003, OR = 0.38, 95% CI = 0.22–0.65), suggesting that rs3865444 C to A substitution is associated with a decreased risk of developing the secondary progressive form of the disease. Interestingly, this protective effect was observed in both HLA-DRB1*15:01-positive and negative cohorts (Supplementary Table S2).

### 3.3. Association of CD33 rs3865444 with Clinical Phenotypes

Linear regression analysis employed to evaluate the relationship between *CD33* rs3865444 and selected clinical parameters revealed no significant association of the polymorphism with AOO (*p* = 0.88) or MSSS (*p* = 0.57) in the whole patient group. Following the stratification according to the *HLA* carrier status, we could observe a tendency towards a later MS onset in rs3865444 AA homozygotes within the HLA-DRB1*15:01-positive patient cohort, while the opposite trend was found in the HLA-DRB1*15:01-negative cohort. However, neither of these findings reached the level of statistical significance (Table 6).

## 4. Discussion

MS is a multifactorial disorder characterized by chronic inflammation and demyelination, which is followed by remyelination attempts to aid in tissue repair and regeneration [2,7]. The phagocytic uptake and removal of myelin debris by CNS-resident microglia and MDM are particularly important for CNS repair as debris accumulation promotes inflammation, impairs axonal regeneration, arrests the differentiation of oligodendrocyte precursor cells, and inhibits remyelination [14,17,53,54]. Deficient clearance of myelin debris may result in the inability to resolve the inflammatory process and promote remyelination, thereby heightening the susceptibility to MS [14,55]. Multiple membrane receptors have been implicated in controlling microglial/macrophage cell functions and phagocytic activity in the brain, including the Siglec family member CD33 [24]. As an immunomodulatory receptor, CD33 participates in homeostatic cellular mechanisms by suppressing innate immune cells following the recognition of specific glycosylation patterns that act as “self-associated molecular patterns” (SAMP). Upon binding of extracellular or cell-surface sialylated glycosphingolipids or glycoproteins to its amino-terminal Ig V-set domain, CD33 triggers inhibitory signals through a cytosolic immunoreceptor tyrosine-based inhibitory motif (ITIM) and an ITIM-like sequence, effectively reducing microglial phagocytic capacity [24,25].

Recently, an association of the AD-linked variant *CD33* rs3865444:C>A with a reduced risk of MS was reported in a Greek case-control study [19], suggesting a shared role for *CD33* in the pathogenesis of both diseases. In the current study, we aimed to validate this association in the Central European Slovak population and further examine the impact of rs3865444 on the age of disease onset and MS severity. Our initial analysis revealed no significant differences in allele or genotype frequencies between MS cases and controls, indicating that rs3865444 does not confer risk or protection for MS. This is in agreement with the largest GWAS study to date, which did not identify any *CD33* SNP as significantly associated with MS [4]. Possible reasons for the observed discrepancy between the studies may include relatively limited sample sizes and statistical power in both our study and that of Siokas et al. [19], inter-population variation in rs3865444 allele frequencies, differences in linkage disequilibrium between the studied SNP and other functional *CD33* variants, differences in MS/control selection criteria, and others [56]. Another explanation could be the modifying effects of other genetic factors. Case-control association studies have traditionally analyzed individual contributions of candidate gene variants to disease risk, thereby neglecting interactive effects between genetic variants, which may be larger or lower than the main effects at the individual loci or even exist without a significant effect of either of them [57]. Given that the HLA-DRB1*15:01 allele is the single strongest genetic risk factor for MS, it is possible that it modulates the expression of MS risk variants via epistasis, making them precluded in GWAS and other studies. Therefore, we were interested in whether the association of *CD33* rs3865444 with MS is affected by this *HLA* allele, which encodes the major histocompatibility complex class II antigen-presenting molecule HLA-DR15. Analysis in cohorts stratified according to the *HLA* carrier status indeed revealed a protective effect of rs3865444:C>A substitution in a subpopulation of individuals carrying HLA-DRB1*15:01, while no significant association was found in subjects negative for this *HLA* allele. In line with the results of the Greek study [19], the protective effect was attributable to the CA genotype, while CC appeared to be the risk-increasing genotype. Furthermore, the interaction SF analysis confirmed a significant statistical epistasis between *CD33* rs3865444 A and HLA-DRB1*15:01 alleles, suggesting that they display an antagonistic interaction in the context of MS risk. On the other hand, no interaction could be found between rs3865444 and sex, indicating that this *CD33* variant exerts similar effects on MS risk in both sexes. In addition to contributing to the risk of developing the disease, rs3865444 may also affect the course of MS. The results of the present study indicate that the minor A allele is associated with a decreased risk of transition of MS to its secondary progressive form, irrespective of the HLA-DRB1*15:01 carrier status. Furthermore, phenotype-genotype analyses in our MS patients showed no evidence for a significant correlation between rs3865444 and the age of disease onset or MSSS. Altogether, these results suggest that the *CD33* polymorphism might play distinct roles in the disease initiation, progression, and its severity.

The rs3865444:C>A SNP is located 372 bp upstream of the *CD33* transcription start site and is indirectly, via linkage disequilibrium with the rs12459419 SNP in exon 2, linked to alterations in splicing efficiency and an enhanced rate of exon 2 skipping. As a result, the protective minor A allele is associated with increased production of the short CD33m isoform lacking the exon 2-encoded ligand-binding Ig V-set domain at the expense of the functional full-length CD33M [27,28,29,30,31,32,33,34,35]. While the wild-type CD33M isoform was shown to repress microglial proliferation, migration, and phagocytosis of cargo [29,36,38], the alternatively spliced CD33m has the opposite effect and is associated with increased microglial and macrophage function, i.e., enhanced proliferation, phagocytosis, and clearance of protein aggregates, cells, or debris [37,38,39]. The effects of CD33 and its gene polymorphisms on microglia/macrophage-mediated phagocytosis have been most extensively studied in AD, a disease characterized by a failure to clear extracellular amyloid-β (Aβ) peptides from the brain [24]. Here, CD33 activity negatively correlates with the uptake of Aβ42 and promotes amyloid pathology [28,29]. *CD33* rs3865444 A allele likely protects from AD by enhancing Aβ clearance and thus decreasing Aβ plaque burden [25,28,29]. These findings suggest that Aβ plaque can dodge microglia-mediated clearance with the help of sialic acid–CD33 interaction [26].

At the moment, we can only speculate that a similar CD33-related mechanism is also at play in MS pathogenesis. Several recent findings may provide support for our hypothesis. As already mentioned, efficient clearance of myelin debris is important to promote remyelination and inhibit inflammation [54]. CD33 may be among the membrane receptors regulating this process, as CD33M expression was shown to negatively correlate with the uptake of different types of cargo, including myelin [36]. As an ITIM-driven immunoreceptor, CD33 is involved in the modulation of cellular functions by counteracting the activatory signaling induced by various immunoreceptor tyrosine-based activation motif (ITAM) receptors, including TREM2 [58]. The phospholipid-sensing receptor TREM2 is highly expressed by microglia and macrophages in active MS lesions and was shown to promote their survival, proliferation, and phagocytic activity in response to demyelination [16,59,60]. Recent findings indicate that TREM2 activation increases myelin debris uptake and degradation as well as the formation of mature oligodendrocytes from precursor cells, resulting in accelerated clearance of debris by microglia and enhanced remyelination and axonal integrity [16]. It is tempting to suggest that lack of inhibitory signaling in the protective CD33m isoform due to loss of its ligand-binding capacity would unblock TREM2 signaling and lead to enhanced phagocytosis and degradation of myelin debris, a key step to allowing remyelination and resolution of inflammation. Recently, an alternative hypothesis for the protective effect of rs3865444 A allele in AD was proposed, suggesting that its association with enhanced phagocytic activity does not stem from decreased expression of functional inhibitory CD33M, but rather due to CD33m being a gain-of-function variant [25]. Here, the loss of the Ig V-set domain would allow CD33m to cluster in a novel conformation and constitutively act as an ITAM to activate microglial function [25]. A follow-up study did provide support for this hypothesis by showing that CD33m has a preference for intracellular location and its association with enhanced phagocytosis is dependent on its cytoplasmic signaling motifs and not due to loss of ligand binding [37]. However, it remains to be elucidated if this mechanism also applies to MS.

Besides its strengths, this study also has several limitations. First, the sample sizes and statistical power of the study were relatively limited, and thus additional analyses in independent populations are warranted to validate our findings. Second, the study focused only on the best known *CD33* polymorphism, thus omitting other variants within or in close proximity of the *CD33* gene which could also contribute to MS risk. Third, although we found a significant statistical epistasis between rs3865444 A and HLA-DRB1*15:01 alleles, we cannot exclude the possibility that other genetic variants also have effects on the association of rs3865444 with MS risk. Fourth, we used MSSS to assess the severity of MS in the current study. This approach, although traditionally used, is not entirely optimal as MSSS may not be a stable measure of long-term outcome and relies on a single EDSS measurement that is prone to inaccuracy [61]. Therefore, future studies should consider survival analysis of long-term disability data.

## 5. Conclusions

This study provides additional evidence for the possible role of the *CD33* rs3865444:C>A SNP as one of the genetic driving forces involved in MS development. Interestingly, the protective effect of the C to A substitution on susceptibility to MS seems to be limited to a subpopulation positive for the major MS risk allele HLA-DRB1*15:01. Furthermore, rs3865444 may also reduce the risk of developing the secondary progressive form of MS, but it does not seem to have an individual impact on the age of disease onset or its severity. Additional studies are required to shed more light on the antagonistic epistasis between *CD33* rs3865444 A and HLA-DRB1*15:01 alleles and fully understand the role of CD33 in MS pathogenesis. Therapeutic strategies targeting CD33 through small molecules, monoclonal antibodies or other approaches are already being considered for the treatment of AD [24] and could potentially provide perspective for MS as well.

## Figures and Tables

**Table 1:** Demographic and clinical characteristics of multiple sclerosis (MS) patients and controls.

Parameter	Controls (*n* = 1145)	MS Total (*n* = 579)	*p*-Value	MS Females	MS Males	*p*-Value
				(*n* = 403)	(*n* = 176)	
Age (years)	51.86 ± 21.09	41.61 ± 10.51	**<0.0001**	41.84 ± 10.46	41.08 ± 10.62	0.26
Age of onset (years)	-	29.68 ± 9.78	-	29.63 ± 9.60	29.80 ± 10.21	0.87
Sex (females/males)	717/428	403/176	**0.0041**	-	-	-
MS course (RR/SP)	-	511/68	-	358/45	153/23	0.51
MS duration (years)	-	11.91 ± 7.13	-	12.19 ± 7.11	11.26 ± 7.15	0.059
EDSS	-	3.56 ± 1.56	-	3.56 ± 1.47	3.58 ± 1.76	0.71
MSSS	-	4.41 ± 2.09	-	4.33 ± 1.97	4.60 ± 2.32	0.19
HLA-DRB1*15:01 positivity	230 (20.09%)	298 (51.47%)	**<0.0001**	214 (53.10%)	84 (47.73%)	0.23

Data are shown as the mean with standard deviation or as number (%). Significant *p*-values are shown in bold. EDSS: Expanded Disability Status Scale; MS: multiple sclerosis; MSSS: Multiple Sclerosis Severity Score; RR: relapsing-remitting; SD: standard deviation; SP: secondary progressive.

**Table 2:** Association between *CD33* rs3865444 and MS in the whole population.

Allele/Genotype	MS (*n* = 579)	Controls (*n* = 1145)	Genetic Model	Logistic Regression Analysis	
				*p*-Value	OR (95% CI)
			Allele contrast (A vs. C)	0.68	0.97 (0.83–1.13)
C	806 (69.60%)	1578 (68.91%)	Codominant (CA vs. CC)	0.45	0.91 (0.73–1.15)
A	352 (30.40%)	712 (31.09%)	Codominant (AA vs. CC)	0.97	1.00 (0.68–1.46)
CC	285 (49.22%)	538 (46.99%)	Dominant (AA + CA vs. CC)	0.52	0.93 (0.75–1.16)
CA	236 (40.76%)	502 (43.84%)	Recessive (AA vs. CA + CC)	0.83	1.04 (0.72–1.50)
AA	58 (10.02%)	105 (9.17%)	Over-dominant (CA vs. CC + AA)	0.43	0.91 (0.73–1.14)
			Log-additive	0.69	0.97 (0.82–1.14)

Allele and genotype counts are presented with frequencies in parentheses. *p*, OR, and 95% CI values for genotype comparisons were adjusted for age, sex, and HLA-DRB1*15:01 carrier status. CI: confidence interval; MS: multiple sclerosis; OR: odds ratio.

**Table 3:** Association between *CD33* rs3865444 and MS in cohorts stratified according to the HLA-DRB1*15:01 carrier status.

Allele/Genotype	MS	Controls	Genetic Model	Logistic Regression Analysis	
				*p*-Value	OR (95% CI)
HLA-DRB1*15:01- **positive cohort**					
			Allele contrast (A vs. C)	0.14	0.82 (0.63–1.07)
C	424 (71.14%)	308 (66.96%)	Codominant (CA vs. CC)	**0.028**	0.64 (0.44–0.95)
A	172 (28.86%)	152 (33.04%)	Codominant (AA vs. CC)	0.62	0.85 (0.44–1.64)
CC	154 (51.68%)	99 (43.04%)	Dominant (AA + CA vs. CC)	**0.040**	0.68 (0.47–0.98)
CA	116 (38.93%)	110 (47.83%)	Recessive (AA vs. CA + CC)	0.89	1.04 (0.55–1.97)
AA	28 (9.39%)	21 (9.13%)	Over-dominant (CA vs. CC + AA)	**0.031**	0.66 (0.46–0.96)
			Log-additive	0.13	0.80 (0.61–1.07)
HLA-DRB1*15:01- **negative cohort**					
			Allele contrast (A vs. C)	0.52	1.07 (0.87–1.31)
C	382 (67.97%)	1270 (69.40%)	Codominant (CA vs. CC)	0.50	1.10 (0.82–1.47)
A	180 (32.03%)	560 (30.60%)	Codominant (AA vs. CC)	0.63	1.10 (0.69–1.77)
CC	131 (46.62%)	439 (47.98%)	Dominant (AA + CA vs. CC)	0.49	1.10 (0.84–1.45)
CA	120 (42.70%)	392 (42.84%)	Recessive (AA vs. CA + CC)	0.81	1.06 (0.67–1.66)
AA	30 (10.68%)	84 (9.18%)	Over-dominant (CA vs. CC + AA)	0.58	1.08 (0.82–1.43)
			Log-additive	0.53	1.07 (0.87–1.31)

Allele and genotype counts are presented with frequencies in parentheses. *p*, OR, and 95% CI values for genotype comparisons were adjusted for age and sex. Significant *p*-values are shown in bold. CI: confidence interval; MS: multiple sclerosis; OR: odds ratio.

**Table 4:** Statistical interaction between *CD33* rs3865444 A and HLA-DRB1*15:01 alleles.

*CD33* rs3865444 A	HLA-DRB1*15:01	MS (*n* = 579)	Controls (*n* = 1145)	Logistic Regression Analysis		SF (*p*-Value)
				*p*-Value	OR (95% CI)	
−	−	131 (22.63%)	439 (38.34%)	reference		**0.62 (0.032)**
+	−	150 (25.91%)	476 (41.57%)	0.49	1.10 (0.84–1.45)	
−	+	154 (26.60%)	99 (8.65%)	**<0.0001**	5.39 (3.87–7.52)	
+	+	144 (24.87%)	131 (11.44%)	**<0.0001**	3.67 (2.68–5.03)	

The “−” sign denotes no copies of the allele, while the “+” sign denotes the presence of at least one copy of the allele. Allele counts are presented with frequencies in parentheses. *p*, OR, and 95% CI values were adjusted for age and sex. Significant *p*-values are shown in bold. SF was calculated as the ratio of the observed OR for both factors combined (3.67) to the predicted OR assuming independent effects of each factor (1.10 × 5.39 = 5.93). CI: confidence interval; MS: multiple sclerosis; OR: odds ratio; SF: synergy factor.

**Table 5:** Comparison of *CD33* rs3865444 allele and genotype distribution between RR-MS, SP-MS, and control groups.

A/G	RR-MS (*n* = 511)	SP-MS	GM	RR-MS vs. C		SP-MS vs. C		SP-MS vs. RR-MS	
		(*n* = 68)		*p*	OR (95% CI)	*p*	OR (95% CI)	*p*	OR (95% CI)
			AC	0.64	1.04 (0.86–1.22)	**0.0032**	0.52 (0.34–0.81)	**0.0023**	0.50 (0.32–0.79)
C	696 (68.10%)	110 (80.88%)	CD1	0.78	1.03 (0.81–1.32)	**0.0005**	0.38 (0.21–0.67)	**0.0072**	0.43 (0.23–0.81)
A	326 (31.90%)	26 (19.12%)	CD2	0.52	1.13 (0.76–1.67)	0.071	0.41 (0.14–1.18)	**0.040**	0.33 (0.11–1.05)
CC	239 (46.77%)	46 (67.65%)	D	0.67	1.05 (0.84–1.32)	**0.0003**	0.38 (0.22–0.65)	**0.0023**	0.41 (0.23–0.74)
CA	218 (42.66%)	18 (26.47%)	R	0.59	1.11 (0.76–1.67)	0.30	0.60 (0.21–1.71)	0.15	0.46 (0.15–1.42)
AA	54 (10.57%)	4 (5.88%)	OD	0.92	1.01 (0.80–1.28)	**0.0016**	0.42 (0.24–0.74)	**0.022**	0.50 (0.27–0.92)
			LA	0.57	1.05 (0.88–1.25)	**0.0007**	0.48 (0.30–0.75)	**0.0028**	0.50 (0.31–0.81)

Allele and genotype counts are presented with frequencies in parentheses. *p*, OR, and 95% CI values for genotype comparisons were adjusted for age, sex, and HLA-DRB1*15:01 carrier status. AC: allele contrast model (A vs. C); A/G: allele/genotype; C: controls; CD1: codominant model (CA vs. CC); CD2: codominant model (AA vs. CC); CI: confidence interval; D: dominant model (AA + CA vs. CC); GM: genetic model; LA: log-additive model; OD: over-dominant model (CA vs. CC + AA); OR: odds ratio; R: recessive model (AA vs. CA + CC); RR-MS: relapsing-remitting multiple sclerosis; SP-MS: secondary progressive multiple sclerosis. Significant *p*-values are shown in bold.

**Table 6:** Association of CD33 rs3865444 with MS phenotypes.

Phenotype	Patient Group	Genotypes			Best Model	*p*-Value
		CC	CA	AA		
	Whole	29.68 ± 9.66	29.62 ± 9.88	29.88 ± 10.09	dominant	0.88 *
AOO	HLA-DRB1*15:01 +	28.60 ± 8.72	28.22 ± 9.22	30.54 ± 11.15	recessive	0.27 ^†^
	HLA-DRB1*15:01 −	30.96 ± 10.56	30.98 ± 10.34	29.27 ± 9.14	recessive	0.36 ^†^
	Whole	4.43 ± 2.12	4.43 ± 2.10	4..27 ± 1.93	recessive	0.57 ^‡^
MSSS	HLA-DRB1*15:01 +	4.51 ± 1.99	4.45 ± 2.10	4.32 ± 1.95	recessive	0.41 ^$^
	HLA-DRB1*15:01 −	4.34 ± 2.26	4.41 ± 2.11	4.23 ± 1.94	dominant	0.72 ^$^

Data are shown as the mean with standard deviation. The “−” sign denotes no copies of the *HLA* allele, while “+” sign denotes the presence of at least one copy of the allele. Linear regression analysis was adjusted for: * sex and HLA-DRB1*15:01 carrier status; ^†^ sex; ^‡^ AOO, sex, and HLA-DRB1*15:01 carrier status; ^$^ AAO and sex. AOO: age of onset; MSSS: Multiple Sclerosis Severity Score.

## Data Availability

The data presented in this study are available on request from the corresponding author.

## Supplementary Materials

The following supporting information can be downloaded at: https://www.mdpi.com/article/10.3390/life12071094/s1, Table S1: Association between *HLA-DRB1*15:01* allele and MS; Table S2: Comparison of *CD33* rs3865444 allele and genotype distribution between RR-MS, SP-MS, and control groups after stratification according to the *HLA-DRB1*15:01* carrier status.
