# Supplementary material for: Alzheimer’s Disease Risk Variant rs3865444 in the CD33 Gene: A Possible Role in Susceptibility to Multiple Sclerosis
Source: Life (Basel). 2022 Jul 21;12(7):1094. doi: 10.3390/life12071094 (PMC9324428; doi:10.3390/life12071094)
Supplement: Supplementary file 1 [file life-12-01094-s001.zip › life-1812612-supplementary.pdf]

Supplementary Table S1. Association between *HLA-DRB1*15:01* allele and MS.

*HLA-DRB1*15:01* allele	MS (*n* = 579)	Controls (*n* = 1145)	Genetic model	Logistic regression analysis	
				p-Value	OR (95% CI)
			Codominant (1 vs. 0 copies)	**<0.0001**	3.77 (2.98–4.76)
0 copies (-/-)	281 (48.53%)	915 (79.91%)	Codominant (2 vs. 0 copies)	**<0.0001**	17.01 (7.73–37.39)
1 copy (+/-)	257 (44.39%)	222 (19.39%)	Dominant (1 + 2 vs. 0 copies)	**<0.0001**	4.23 (3.37–5.30)
2 copies (+/+)	41 (7.08%)	8 (0.70%)	Recessive (2 vs. 0 + 1 copies)	**<0.0001**	10.98 (5.02–24.01)
			Over-dominant (1 vs. 0 + 2 copies)	**<0.0001**	3.30 (2.63–4.15)
			Log-additive	**<0.0001**	3.85 (3.13–4.74)

Copy numbers of *HLA-DRB1*15:01* alleles are presented with frequencies in parentheses. *P*, OR, and 95% CI values were adjusted for age and sex. CI: confidence interval; MS: multiple sclerosis; OR: odds ratio.

Supplementary Table S2. Comparison of *CD33* rs3865444 allele and genotype distribution between RR-MS, SP-MS, and control groups after stratification according to the *HLA-DRB1*15:01* carrier status.

A/G	RR-MS	SP-MS	GM	RR-MS vs. C		SP-MS vs. C		SP-MS vs. RR-MS	
				p	OR (95% CI)	*p*	OR (95% CI)	*p*	OR (95% CI)
***HLA-DRB1*15:01*-positive cohort**									
			AC	0.22	0.84 (0.64–1.11)	0.18	0.70 (0.41–1.19)	0.49	0.83 (0.49–1.41)
C	363 (70.62%)	61 (74.39%)	CD1	**0.049**	0.66 (0.44–1.00)	**0.044**	0.48 (0.23–1.00)	0.27	0.62 (0.28–1.37)
A	151 (29.38%)	21 (25.61%)	CD2	0.53	0.81 (0.41–1.62)	0.63	0.77 (0.24–2.46)	0.42	0.61 (0.17–2.14)
CC	130 (50.58%)	24 (58.54%)	D	0.061	0.69 (0.47–1.02)	0.062	0.53 (0.27–1.04)	0.20	0.62 (0.30–1.29)
CA	103 (40.08%)	13 (31.71%)	R	0.97	0.99 (0.51–1.91)	0.92	1.06 (0.34–3.29)	0.63	0.75 (0.22–2.50)
AA	24 (9.34%)	4 (9.76%)	OD	0.062	0.69 (0.47–1.02)	**0.05**	0.50 (0.25–1.02)	0.31	0.68 (0.31–1.45)
			LA	0.15	0.80 (0.60–1.08)	0.16	0.68 (0.39–1.17)	0.24	0.72 (0.41–1.26)
***HLA-DRB1*15:01*-negative cohort**									
			AC	0.10	1.19 (0.97–1.47)	**0.0007**	0.23 (0.09–0.58)	**0.0002**	0.19 (0.08–0.50)
C	333 (65.55%)	49 (90.74%)	CD1	0.078	1.31 (0.96–1.78)	**0.0017**	0.25 (0.09–0.66)	**0.0055**	0.24 (0.08–0.71)
A	175 (34.45%)	5 (9.26%)	CD2	0.22	1.34 (0.83–2.17)	N/A	N/A	N/A	N/A
CC	109 (42.91%)	22 (81.48%)	D	0.062	1.32 (0.99–1.76)	**0.0003**	0.20 (0.08–0.55)	**0.0014**	0.20 (0.07–0.59)
CA	115 (45.28%)	5 (18.52%)	R	0.48	1.18 (0.75–1.86)	N/A	N/A	N/A	N/A
AA	30 (11.81%)	0 (0.00%)	OD	0.15	1.24 (0.93–1.65)	**0.0062**	0.29 (0.11–0.78)	**0.018**	0.30 (0.10–0.87)
			LA	0.085	1.21 (0.98–1.49)	**0.0002**	0.22 (0.09–0.57)	**0.0007**	0.22 (0.08–0.60)

Allele and genotype counts are presented with frequencies in parentheses. *P*, OR, and 95% CI values for genotype comparisons were adjusted for age and sex. AC: allele contrast model (A vs. C); A/G: allele/genotype; C: controls; CD1: codominant model (CA vs. CC); CD2: codominant model (AA vs. CC); CI: confidence interval; D: dominant model (AA + CA vs. CC); GM: genetic model; LA: log-additive model; N/A: not applicable; OD: over-dominant model (CA vs. CC + AA); OR: odds ratio; R: recessive model (AA vs. CA + CC); RR-MS: relapsing-remitting multiple sclerosis; SP-MS: secondary progressive multiple sclerosis.
